# Deletion of a *Hand1* lncRNA-Containing Septum Transversum Enhancer Alters lncRNA Expression but Is Not Required for *Hand1* Expression

**DOI:** 10.3390/jcdd8050050

**Published:** 2021-05-04

**Authors:** Rajani M. George, Anthony B. Firulli

**Affiliations:** Herman B Wells Center for Pediatric Research, Departments of Pediatrics, Anatomy and Medical and Molecular Genetics, Indiana Medical School, Indianapolis, IN 46202, USA; rmgeorge@iu.edu

**Keywords:** HAND1, transcription, heart, epicardium, septum transversum, cardiac morphogenesis, lncRNA

## Abstract

We have previously identified a *Hand1* transcriptional enhancer that drives expression within the septum transversum, the origin of the cells that contribute to the epicardium. This enhancer directly overlaps a common exon of a predicted family of long non-coding RNAs (lncRNA) that are specific to mice. To interrogate the necessity of this *Hand1* enhancer, as well as the importance of these novel lncRNAs, we deleted the enhancer sequences, including the common exon shared by these lncRNAs, using genome editing. Resultant homozygous *Hand1* enhancer mutants (*Hand1**^Δ^**^ST/**Δ**ST^*) present with no observable phenotype. Assessment of lncRNA expression reveals that *Hand1**^Δ^**^ST/**Δ**ST^* mutants effectively eliminate detectable lncRNA expression. Expression analysis within *Hand1**^Δ^**^ST/**Δ**ST^* mutant hearts indicates higher levels of *Hand1* than in controls. The generation of *Hand1* compound heterozygous mutants with the *Hand1^LacZ^* null allele (*Hand1**^Δ^**^ST/LacZ^*) also did not reveal any observable phenotypes. Together these data indicate that deletion of this *Hand1* enhancer and by consequence a family of murine-specific lncRNAs does not impact embryonic development in observable ways.

## 1. Introduction

The epicardium, the outer covering of the developing heart is a dynamic structure that contributes to cardiac myofibroblasts, coronary smooth muscle, adipocytes and possibly some coronary endothelium within the mature heart [1]. Epicardial cells originate from an embryonic structure termed the proepicardial organ (PEO), which itself is derived from cells that originate within the anterior portion of the septum transversum (ST) [2,3,4]. The ST is a transient embryonic structure that arises from a folding of the lateral mesoderm caudal to the developing heart in mammals and gives rise to structures that physically separate the abdominal and thoracic cavities [5]. In mouse E9.0 embryos, cells that originate from the anterior ST undergo epithelial to mesenchymal transition (EMT), move into the PEO, and begin to migrate over the outer surface of the developing heart giving rise to the epicardium. By E10.5 the developing heart is completely covered by epicardial cells. At E12.5, a subset of epicardial cells undergo a successive round of EMT and move into the underlying myocardium and it is these epicardial derived cells which differentiate further into cardiac myofibroblasts, coronary smooth muscle, cardiac adipocytes, and possibly coronary endothelial cells [6,7,8,9].

The basic helix loop helix (bHLH) transcription factors HAND1 and HAND2 are required for cardiogenesis and have been implicated in epicardial formation. [10,11,12]. Lineage trace analysis reveals that *Hand1*-lineage marks cells within the anterior ST, PEO, epicardium and its secondary EMT derivatives; however, *Hand1* expression is only detectable within the anterior ST [13]. *Hand2* expression is observed within the PEO and epicardium [13]. Deletion of *Hand2* using *WT1^ERT2Cre^* [14], which is expressed specifically in the epicardium during development, results in a similar phenotype as the deletion of *Hand2* within the *Hand1*-lineage, malformed coronaries [13].

Interrogation of the conserved non-coding DNA regulatory elements upstream of the *Hand1* transcriptional start site has identified an enhancer element that drives *LacZ* reporter expression within the anterior ST [15]. Interestingly, a family of predicted murine-specific lncRNAs overlaps this ST enhancer. A common exon shared within this lncRNA family resides within the ST enhancer sequences. To determine the function of this *Hand1* enhancer and the possible role of these novel predicted lncRNAs in the formation of the PEO and epicardium, we employed CRISPR/Cas9 to delete the ST enhancer and subsequently the lncRNAs. Results show that we obtained 6 *Hand1**^Δ^**^ST^* lines of insertion-deletion (indels) which span a range of 1795 to 1786 base pairs of deletion. The largest deletion (1795 bp) was bred to homozygosity-producing viable mice and used for all subsequent experiments. *Hand1* expression is observed to be elevated within the heart; however, assessment of PEO, epicardial and epicardial derivative marker gene expression reveal no significant changes supporting the observation that epicardium and epicardial derived structures appear normal. Compound intercross of the *Hand1**^Δ^**^ST^* with the *Hand1^LacZ^* null allele [11] did not introduce any notable phenotypes. Expression analysis confirms that the lncRNA family expression is compromised within *Hand1**^Δ^**^ST/**Δ**ST^* embryos. Thus, although this *Hand1* enhancer drives ST-specific expression, it is not necessary for the formation of the PEO or epicardium nor does it reduce expression within the ST, suggesting additional unidentified enhancers can compensate for its loss. Finally, the novel murine-specific lncRNAs, when deleted do not reveal an observable functional role.

## 2. Materials and Methods

### 2.1. Mouse Strains and Genotyping

*Hand1^ΔST^* mice were generated using CRISPR/Cas9 by the Washington University Genome Engineering (St. Louis, MO, USA) and iPSC core in the FVB background. 6 founder lines were screened for enhancer knockout. Lines with spurious insertions were identified by PCR isolating, TOPO cloning, and sequencing the enhancer region. The largest knockout without artifact (1795 bp deletion) was used for subsequent experiments. For controls, FVB mice were maintained as a separate line for *Hand1^+/+^* controls. Genotyping was carried out using Southern blots as previously described [16]. *BamHI* genomic DNA digestion produces an RFLP of 1.6 kb for the Hand1^ΔST^ allele and 3.6 kb for the wildtype allele. Homozygous *Hand1^ΔST/ΔST^* mice were used for timed matings.

### 2.2. Histology

Embryos were fixed in 4% paraformaldehyde, dehydrated, embedded, sectioned, and hematoxylin and eosin (H and E) stained as described [16].

### 2.3. Cloning

Southern blot probe for the *Hand1^ΔST^* allele was amplified and cloned from genomic DNA obtained from FVB mice using Forward 5’-TCGCTGGTTTCTAGCTGTGA-3′ and Reverse 5′-CAGCCCAAATTGCCAGACAC-3′primer sequences. PCR products were cloned into a pCRII-TOPO backbone (ThermoFisher, Waltham, MA, USA). Plasmids were sequenced, digested with *EcoRI*, gel purified, and radiolabeled with α^32^P dCTP using Prime-a-Gene Labeling System (Promega, Madison, WI, USA) to use as probe for Southern blots.

### 2.4. In-Situ Hybridization

Section in-situ hybridizations (ISH) were performed on 10-μm paraffin sections as described previously [16,17]. Whole mount in-situ hybridizations were performed using E10.5 day embryos as described previously [16]. Antisense digoxygenin-labeled riboprobes were synthesized using T7, T3, or SP6 polymerases (Promega) and DIG-Labeling Mix (Roche, Basel, Switzerland) using the following plasmid templates: *Hand1, Tbx18, Tcf21*, and *Postn.*

### 2.5. Quantitative Real Time PCR

Total RNA was isolated from E9.5 hearts or torsos not including the heart, or E10.5 hearts alone using the High Pure RNA Isolation Kit (Roche). RNA was used to synthesize cDNA using the High-Capacity cDNA Reverse Transcription Kit (Applied Biosystems, Waltham, MA, USA). For qRT-PCR, cDNA was amplified using TaqMan Probe-Based Gene Expression Assays (Applied Biosystems) to quantify *Hand1, Tcf21,* and *Tbx18* expression in E9.5 hearts and torsos. Custom primers specific to ST enhancer sequence and putative lncRNAs were synthesized by Integrated DNA Technologies (IDT) P1[F]5’-TGCCGCCGCACGTCTCTAAT-3′ P1[R]5’-ATCCACAGGGCTGCCCTATC-3′ P2[F]5’-AGCTCCTTGAGGCCAGGGAG-3′ P2[R]5’-TAATAGGCAGGAGGTCAATCCCTC-3′ P3[F]5′-AGCAGAGTCTCACTGAACCATACTCCACC-3′ P3[R]5′-AGAGTTGGTTGACCATGTGAGTTATGTGTGAACC-3′. cDNA in E10.5 day hearts was assayed using PowerUp SybrGreen Master Mix (ThermoFisher). qRT-PCR reactions were run on the QuantStudio 3 Real-Time PCR System (ThermoFisher). Normalization to Glyceraldehyde 3-phosphate dehydrogenase (GAPDH) was used to determine relative gene expression and statistical analysis was automatically applied by the instrument software. Significance of qRT-PCR results was determined by a two-tailed student’s t-test followed by post hoc Benjamini–Hochberg FDR correction as automatically calculated by the QuantStudio 3 qRT-PCR thermal cycler software analysis package.

### 2.6. ATAC-Seq

Assay for Transposase-Accessible Chromatin by sequencing was done as previously described [18]. In brief, timed matings were used to generate E10.5 embryos. Hearts were excised in PBS, and single-cell suspension was obtained using the Wheaton tight douncer, and 50,000 cells collected. Cells were lysed in 10 mM Tris pH 7.4, 10 mM NaCl, 3 mM MgCl_2_ and 0.1% IGEPAL CA-630 for 5 min on ice. Nuclei were pelleted and 50 μL tagmentation reaction and subsequent sequencing carried out as per manufacturer’s recommendations (Illumina, San Diego, CA, USA).

## 3. Results

### 3.1. CRISPR/Cas9 Mediated Deletion of the ST Enhancer

**Figure 1:**
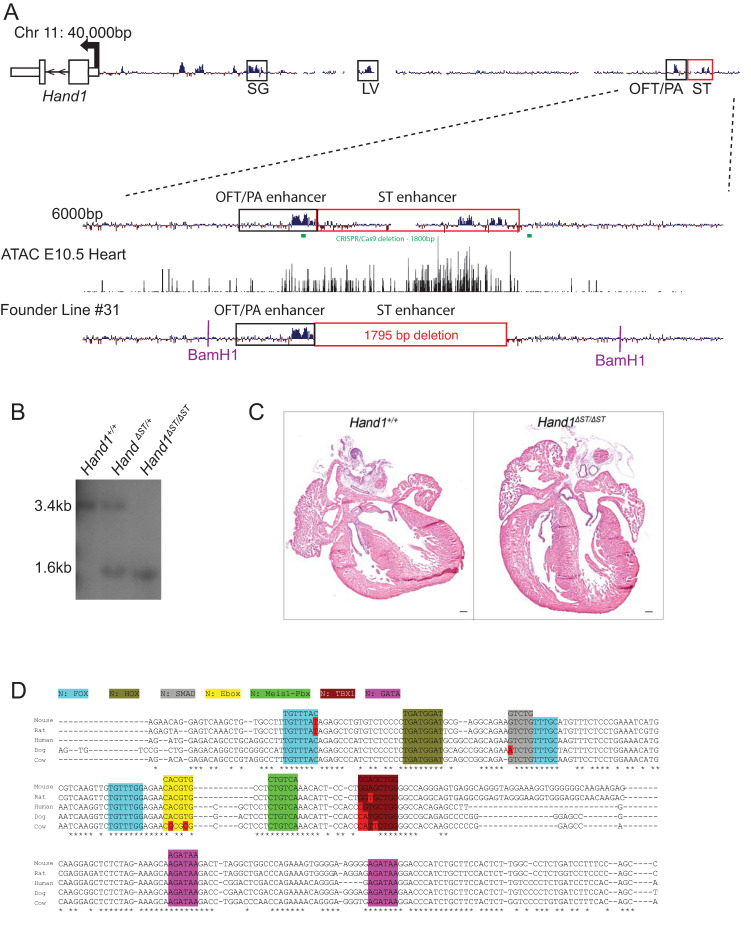
CRISPR/Cas9 mediated deletion of ST enhancer. (**A**) Mammalian conservation track at the mouse Hand1 locus. Conserved non-coding regions with spatial and temporal specific activity are boxed. ST enhancer is boxed in red. Close up region of OFT and ST enhancer are indicated with overlying ATAC-seq from E10.5 hearts. Bars in green represent guide RNA sequences designed for CRISPR/Cas9 mediated deletion of ST enhancer. The largest deletion line used for further analysis with 1795 bp deletion is shown. Representative BamH1 digest sites for Southern blot analysis are indicated. Location of Southern blot probe is indicated. (**B**) Southern blot analysis for genotyping *Hand1****^Δ^****^ST/**Δ**ST^*. Wildtype *Hand1^+/+^*, Heterozygote *Hand1***^Δ^**^ST/+^, and Homozygote *Hand1****^Δ^****^ST/**Δ**ST^* are shown. The *Hand1^+^* allele is 3.6 kb in length and the *Hand1****^Δ^****^ST^* allele is 1.6 kb in length. (**C**) Post-natal day 1 hearts from *Hand1^+/+^ Hand1****^Δ^****^ST/**Δ**ST^* mice. 10μm wax sections are stained with hemotoxylin and eosin. Scale bar 100 μm. (**D**) ClustalW pile up for core conserved region of ST enhancer comparing mouse, rat, human, cow, and dog sequences. Consensus DNA sequence for transcription factor binding sites is indicated. LV, left ventricle; OFT, outflow tract; PA, pharyngeal arches; SG, sympathetic ganglia; ST, septum transversum; ATAC-seq, assay for transposase-accessible chromatin using sequencing.

Previous work has identified a putative *cis*-regulatory enhancer element 34 kb upstream of transcriptional start site of *Hand1* coding sequence marking the ST at embryonic day (E)10.5 (Figure 1A, red box) [15]. ATAC-seq (Assay for Transposase-Accessible Chromatin by sequencing) in E10.5 hearts shows accessible chromatin at this genomic locus at this time point. In order to further characterize this enhancer, we used CRISPR/Cas9 mediated deletion of the 1.8 kb element located at genomic region chr11:57,678,478-57,680,329 (mm9). Guide RNAs (Figure 1A, green bars) were designed for this region and 6 founder lines were generated. These lines were screened for deletion size, and presence of spurious insertions at the locus by PCR amplification, TOPO cloning of insert, and sequencing. The largest deletion without artifact was selected for further breeding. This line, with a 1795 bp deletion, generated viable, phenotypically normal, homozygous *Hand1**^Δ^**^ST/**Δ**ST^* mice as determined by Southern blotting (Figure 1B). These mice were maintained on an FVB background and control *Hand1^+/+^* mice were generated by breeding wildtype FVB mice. *Hand1^+/+^* wildtype allele was detected as a 3.6 kb fragment, and the *Hand1**^Δ^**^ST/**Δ**ST^* enhancer knockout allele was a 1.6 kb fragment on the Southern blot (Figure 1B). *Hand1**^Δ^**^ST/**Δ**ST^* mice were bred to homozygosity and did not show any apparent phenotype. Neonates were obtained at expected ratios and histological analysis of post-natal day 1 *Hand1**^Δ^**^ST/**Δ**ST^* hearts did not show any defects when compared to *Hand1^+/+^* controls (Figure 1C). ClustalW alignment across mammals, comparing mouse sequence to human, rat, cow, and dog shows regions of conservation in consensus transcription factor binding sites (Figure 1D).

### 3.2. Loss of a Hand1 Allele over the ST Enhancer Knockout Doesnot Lead to Embryonic Lethality

**Figure 2:**
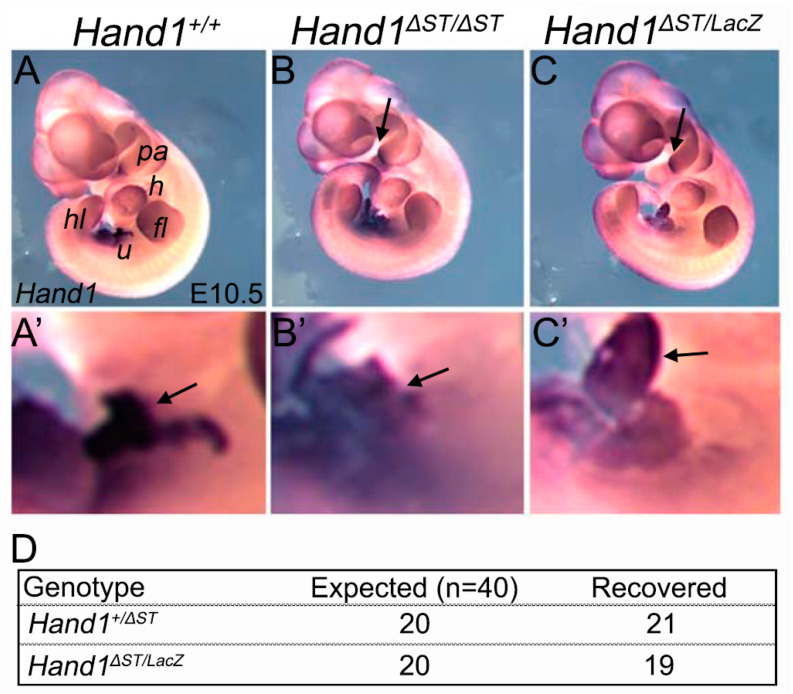
Loss of a *Hand1* Allele over the ST Enhancer knockout does not lead to embryonic lethality. (**A**–**C**) Whole mount in-situ hybridization (WISH) at E9.5 for Hand1 mRNA in Hand1^+/+^, Hand1**^Δ^**^ST/**Δ**ST^, and Hand1^ΔST/LacZ^ embryos showing no qualitative or spatial change in Hand1 mRNA levels. Arrows indicating pharyngeal arch expression of Hand1 in Hand1**^Δ^**^ST/**Δ**ST^ and Hand1**^Δ^**^ST/LacZ^ embryos. *N* = 5 embryos for all genotypes. pa, pharyngeal arches; h, heart; fl, forelimb; hl, hindlimb; u, umbilicus. (**A**’–**C**’) Umbilicus region expression of Hand1 in WISH embryos. (**D**) Table showing expected and recovered genotypes at postnatal day 10 from Hand1**^Δ^**^ST/**Δ**ST^ and Hand1^LacZ/+^ parental crosses.

Since *Hand1**^Δ^**^ST/**Δ**ST^* mice do not demonstrate any observable phenotype, we crossed homozygous *Hand1**^Δ^**^ST/**Δ**ST^* to the *Hand1^LacZ/+^* mouse [11] to determine if loss of one functional *Hand1* allele resulted in a more deleterious phenotype. Embryos from the resulting cross were subjected to whole mount in-situ hybridization (WISH) at E10.5 to determine spatial or qualitative changes in *Hand1* gene expression levels. Comparison of *Hand1^+/+^* controls to *Hand1**^Δ^**^ST/LacZ^* or *Hand1**^Δ^**^ST/**Δ**ST^* embryos showed no appreciable difference in *Hand1* expression (Figure 2A–C). Since the OFT/PA enhancer is directly 3′ of the ST enhancer [15], we carefully examined PA expression of *Hand1* within *Hand1**^Δ^**^ST/**Δ**ST^* embryos. Results reveal that *Hand1* PA expression is maintained within *Hand1**^Δ^**^ST/**Δ**ST^* embryos (arrow, Figure 2B) as well as within *Hand1**^Δ^**^ST/LacZ^* embryos (arrow, Figure 2C). We also examined expression within the umbilical region just caudal to the heart and observed no significant changes in *Hand1* expression (arrows, Figure 2A’–C’) Analysis of mendelian ratios from breeding *Hand1**^Δ^**^ST/**Δ**ST^* and *Hand1^LacZ/+^* mice (*n* = 40) show *Hand1**^Δ^**^ST/LacZ^* pups are encountered at expected frequency when genotyped at postnatal day 10, and these mice are viable and fertile (Figure 2D).

### 3.3. ST and PEO In Hand1**^Δ^**^ST/**Δ**ST^ Embryo

**Figure 3:**
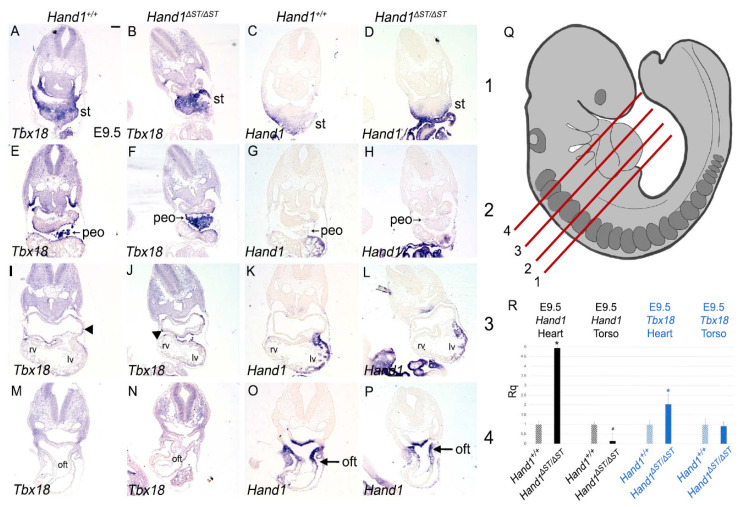
ST And PEO In *Hand1**^Δ^**^ST/**Δ**ST^* Embryo. (**A**–**P**) Section *in-situ* hybridization at E9.5 for *Tbx18* and *Hand1* mRNA in *Hand1^+/+^* and *Hand1****^Δ^****^ST/^****^Δ^****^ST^* embryos. Arrowheads indicate *Tbx18* expressing cells migrating over the heart to form the epicardium. Arrows indicate *Hand1* expression in the outflow tract. st, septum transversum; peo, proepicardial organ; rv, right ventricle; lv, left ventricle; oft, outflow tract. *n*=6 for all genotypes. Scale bar 100μm. (**Q**) Representation of E9.5 embryo showing planes of section indicated at rows: 1 (st), 2 (peo), 3 (four chamber heart), 4 (developing outflow tract). (R) qRT-PCR at E9.5 for *Hand1* and *Tbx18* expression in heart, and torso (dissected to exclude the heart, but with PEO and ST intact) in *Hand1^+/+^* and *Hand1****^Δ^****^ST/**Δ**^^ST^* embryos. *n* = 9 embryos, pooled. Significant increase in *Hand1* (* indicates *p* ≤ 0.01, # indicates *p* ≤ 0.05) and *Tbx18* (* indicates *p* ≤ 0.01) are observed. Error bars represent the high and low range of replicate cycle reads within each primer set.

In order to carefully identify the ST and PEO within *Hand1**^Δ^**^ST/**Δ**ST^* embryos, we interrogated expression of transcription factor *Tbx18*. *Tbx18* expression marks the ST, the PEO, and epicardial cells laminating over the cardiac surface at E9.5 (Figure 3A,E,I) [19,20]. *Hand1* is expressed within the ST but not the PEO (Figure 3C,G) [13]. To test if ST and/or PEO development within *Hand1**^Δ^**^ST/**Δ**ST^* embryos is affected by loss of the ST enhancer, E9.5 embryos were transverse sectioned and serial sections used for ISH to detect *Hand1* and *Tbx18* mRNA. Analysis of mRNA expression in *Hand1**^Δ^**^ST/**Δ**ST^* embryos shows *Tbx18* expression within the ST of *Hand1**^Δ^**^ST/**Δ**ST^* embryos is similar to that observed in *Hand1^+/+^* controls (Figure 3A,B). Surprisingly, *Hand1* expression within the ST appears indistinguishable when comparing *Hand1**^Δ^**^ST/**Δ**ST^* and *Hand1^+/+^* embryos (Figure 3C,D), suggesting that *Hand1* expression within the ST is regulated by redundant enhancers.

Cells within the PEO are identified by *Tbx18* expression and their characteristic rounded cellular morphology at E9.5 as they migrate to envelop the developing heart (Figure 3E) [21]. *Hand1* expression is not observed within the PEO, as has been previously reported (Figure 3G) [13]. In *Hand1**^Δ^**^ST/**Δ**ST^* embryos at a similar plane of section, we observe PEO cells that express *Tbx18* (Figure 3F). In both *Hand1^+/+^* control and *Hand1**^Δ^**^ST/**Δ**ST^* embryos, *Tbx18* expressing cells are observed laminating over the heart to give rise to the epicardium (arrowheads, Figure 3I,J). *Hand1* expression within the left ventricle (LV) and outflow tract (OFT) appear unchanged within *Hand1**^Δ^**^ST/**Δ**ST^* embryos compared to *Hand1^+/+^* controls (Figure 3K,L,O,P arrow). In order to quantify any changes in gene expression, *Hand1**^+^**^/+^* and *Hand1**^Δ^**^ST/**Δ**ST^* embryos at E9.5 are dissected to separate the heart from the torso and are pooled for qRTPCR using Taqman probes against *Hand1* and *Tbx18*. Surprisingly, results show that *Hand1* expression within *Hand1**^Δ^**^ST/**Δ**ST^* hearts is significantly upregulated (4.935 fold, *p* = 0.001) as compared to *Hand1^+/+^* controls (Figure 3R). *Hand1* expression levels within the torso, excluding the heart, but including the ST and PEO, are significantly decreased in *Hand1**^Δ^**^ST/**Δ**ST^* compared to *Hand1**^+^**^/+^* (0.143 fold, *p* = 0.034) (Figure 3R). *Tbx18* expression in E9.5 hearts is increased by a modest but significant (2.026 fold, *p* = 0.001) level within *Hand1**^Δ^**^ST/**Δ**ST^* compared to *Hand1^+/+^* controls (Figure 3R). *Tbx18* expression within *Hand1**^+^**^/+^* and *Hand1**^Δ^**^ST/**Δ**ST^* torsos remains unchanged (0.909 fold, *p* = 0.368) (Figure 3R).

### 3.4. Expression of Hand2 in Hand1^ΔST/ΔST^ Embryos

**Figure 4:**
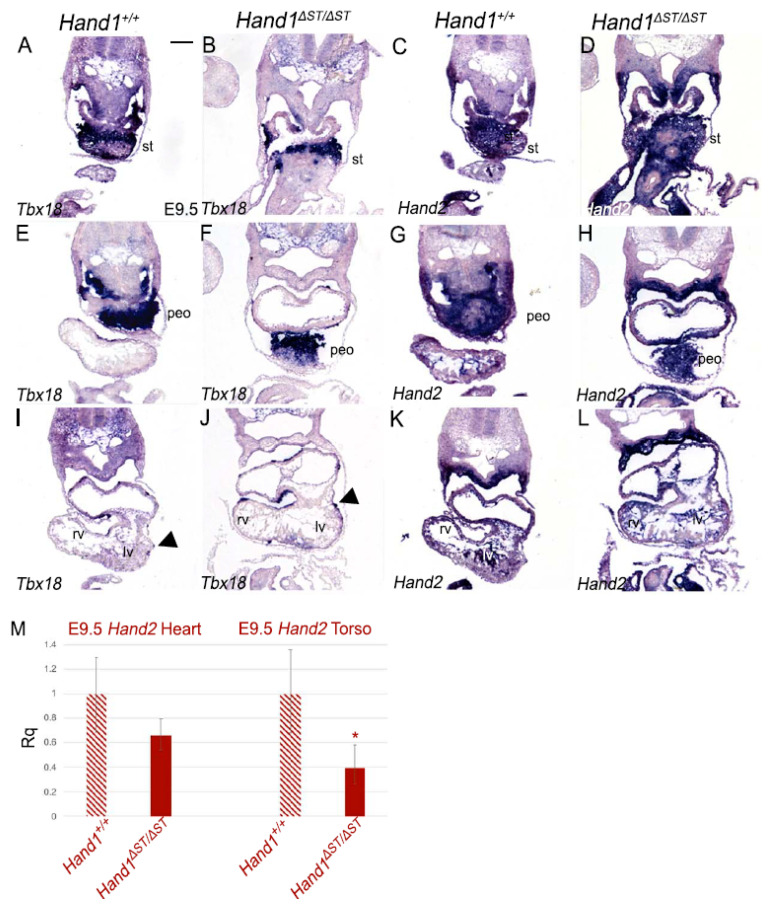
*Hand2* expression in *Hand1****^Δ^****^ST/**Δ**ST^* Embryos. (**A**–**L**) Section in-situ hybridization at E9.5 for *Tbx18* and *Hand2* mRNA in *Hand1^+/+^* and *Hand1****^Δ^****^ST/**Δ**ST^* embryos. Arrowheads indicate *Tbx18* expressing cells migrating over the heart to form the epicardium. st, septum transversum; peo, proepicardial organ; rv, right ventricle; lv, left ventricle. *n* = 3 for all genotypes. Scale bar 100 μm. (**M**) qRT-PCR at E9.5 for *Hand2* expression in heart, and torso (dissected to exclude the heart, but with PEO and ST intact) in *Hand1^+/+^* and *Hand1****^Δ^****^ST/**Δ**ST^* embryos (* indicates *p* ≤ 0.01). *n* = 9 embryos, pooled. Error bars represent the high and low range of replicate cycle reads within each primer set.

Hand1 and Hand2 are expressed in overlapping domains during heart development [12]. Previous work has also suggested functional redundancy in function of these factors [13,22]. To determine if loss of the ST enhancer results in changes in Hand2 expression, E9.5 embryos were sectioned and Hand1**^+^**^/+^ and Hand1^ΔST/ΔST^ embryos were compared (Figure 4). As previously reported, Hand2 is robustly expressed in the ST (Figure 4A,C) and PEO (Figure 4E,G) [13]. Hand2 domain of expression in Hand1^ΔST/ΔST^ embryos compared to Hand1**^+^**^/+^ in ST (Figure 4B,D) and PEO (Figure 4F,H) appears unchanged. In the four-chambered heart, Hand2 expression appears unchanged between Hand1**^+^**^/+^ and Hand1^ΔST/ΔST^ embryos (Figure 4K,L). Loss of the ST enhancer in Hand1^ΔST/ΔST^ embryos does not appear to change the domain or qualitative level of Hand2 expression in the ST or its derivatives. qRTPCR analysis using Taqman probes against Hand2 in pooled E9.5 embryos show Hand2 expression levels as trending lower but not significantly different within Hand1^ΔST/ΔST^ compared to Hand1**^+^**^/+^ hearts (0.657 fold, *p* = 0.083) (Figure 4M). Hand2 expression within the torso, similar to Hand1, is significantly decreased (0.390 fold, *p* = 0.021) in Hand1^ΔST/ΔST^ compared to Hand1**^+^**^/+^ embryos (Figure 4M).

### 3.5. Development of the Epicardium Is Unaffected In Hand **^Δ^**^ST/**Δ**ST^ Embryo

**Figure 5:**
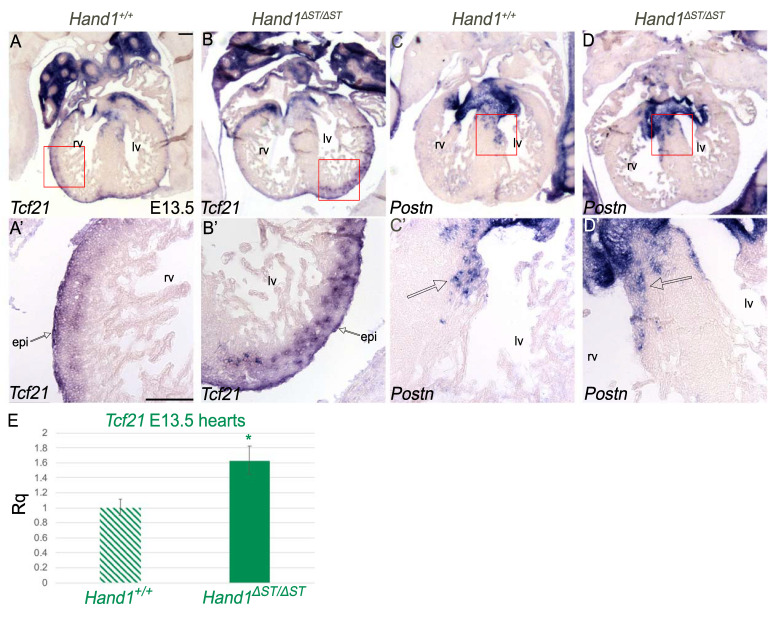
Development of The Epicardium is Unaffected in *Hand1**^Δ^**^ST/**Δ**ST^* Embryo. (**A**,**B**) and (**A**’,**B**’) Section in-situ hybridization at E13.5 *Hand1^+/+^* and *Hand1^Δ^^ST/ΔST^* embryos for *Tcf21* showing four chamber view and at higher magnification. Arrow indicating epicardium. (**C**,**D**) and (**C**’,**D**’) Section in-situ hybridization at E13.5 *Hand1^+/+^* and *Hand1^Δ^^ST/ΔST^* embryos for *Periostin* (*Postn*) showing four chamber view and at higher magnification. Arrow indicating cardiac fibroblasts. rv, right ventricle; lv, left ventricle; epi, epicardium. Scale bar 100 μm. (**E**) qRT-PCR at E13.5 for *Tcf21* in *Hand1^+/+^* and *Hand1^Δ^^ST/ΔST^* hearts. Significant increase in *Tcf21* (* indicates *p* ≤ 0.01) is observed. Error bars represent the high and low range of replicate cycle reads within each primer set.

During heart development, the ST is the source of cells that gives rise to the epicardium [1]. By E13.5, epicardial cells cover the four-chambered heart and are marked by expression of *Tcf21* [23]. To determine if loss of the ST enhancer results in changes in epicardial development, E13.5 *Hand1**^+^**^/+^* and *Hand1**^Δ^**^ST/**Δ**ST^* embryos were sectioned. Comparison of *Tcf21* expression in *Hand1**^+^**^/+^* (Figure 5A,A’) and *Hand1**^Δ^**^ST/**Δ**ST^* (Figure 5B,B’) epicardium suggests that epicardial development in *Hand1**^Δ^**^ST/**Δ**ST^* embryos is normal. Higher power views of *Tcf21* expression at the epicardial surface (red square Figure 4A,B) reveal infiltration of *Tcf21* positive epicardial cells within myocardium indicating the expected secondary EMT observed in normal development (Figure 5A’,B’). qRT-PCR analysis for *Tcf21* using Taqman probes in E13.5 hearts suggests a modest but significant increase (1.626 fold, *p* = 0.005) in *Tcf21* transcript in *Hand1**^Δ^**^ST/**Δ**ST^* hearts compared to *Hand1**^+^**^/+^* controls (Figure 5E).

Epicardial cells undergo secondary EMT to give rise to epicardial derived cells (EPDCs) that move into the myocardium and eventually differentiate into cardiac fibroblasts that contribute to the developing valves [7]. *Periostin* (*Postn*) is expressed by these EPDCs [24]. In order to confirm that loss of ST enhancer appears not to affect EPDC development, section ISH was carried out at E13.5 to examine *Postn* expression within *Hand1**^+^**^/+^* and *Hand1**^Δ^**^ST/**Δ**ST^* embryos (Figure 5C,D). Comparison of *Hand1**^+^**^/+^* and *Hand1**^Δ^**^ST/**Δ**ST^* embryos (Figure 5C’,D’ respectively) suggests an increased region of *Postn* expression in *Hand1**^Δ^**^ST/**Δ**ST^* embryos. Given that *Tcf21* expression is upregulated within *Hand1**^Δ^**^ST/**Δ**ST^* embryos along with the increased expression of *Postn*, our data suggests an expansion of EPDCs in *Hand1**^Δ^**^ST/**Δ**ST^* embryos.

### 3.6. Loss of Putative LncRNA Family Does Not Affect Heart Development

**Figure 6:**
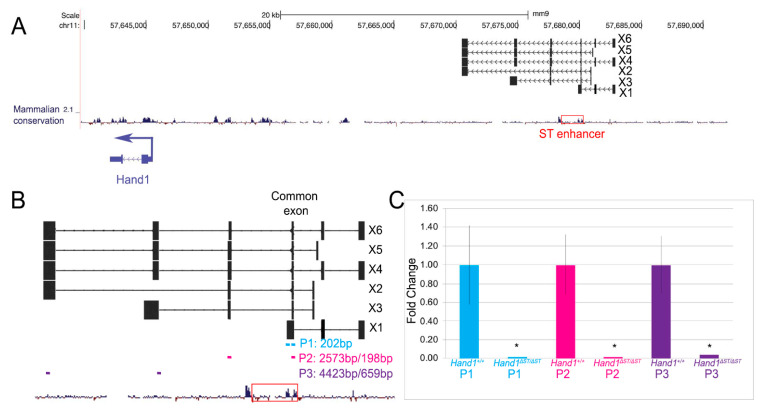
Loss of Putative LncRNA Family does not Affect Heart Development. (**A**) Schematic showing predicted lncRNAs X1, X2, X3, X4, X5, X6 upstream of *Hand1* coding region. Red box indicating ST enhancer deletion. (**B**) Schematic showing lncRNA overlaid with ST enhancer. Common exon shared between all lncRNAs that overlaps with ST enhancer is marked. Two primer sets designed to detect lncs P1 (blue, 202 bp lncRNA product size), P2 (magenta, 2573 bp genomic product size, 198 bp lncRNA product size), P3 (purple, 4423 bp genomic product size, 659 bp lncRNA product size) are marked. (**C**) qRT-PCR results to detect lncRNAs in E10.5 *Hand1^+/+^* and *Hand1^Δ^^ST/ΔST^* embryos. (* indicates *p* ≤ 0.05). Error bars indicate standard error.

The mouse *Hand1* locus contains 6 putative lncRNAs (X1–X6) predicted by Gnomon (NCBI eukaryotic gene prediction tool) to be upstream of the *Hand1* transcriptional start site, that transcribe in the same direction as *Hand1*, and partially overlap with the ST enhancer (Figure 6A, red box). Primers designed internally to a common exon shared by all 6 of the putative lncRNAs (Figure 6B, P1, blue) detect lncRNA transcript within E10.5 *Hand1**^+^**^/+^* hearts, with no expression observed in *Hand1**^Δ^**^ST/**Δ**ST^* hearts (Figure 6C). Primers that are intron spanning (Figure 6B, P2, magenta) also reveal lncRNA expression within E10.5 *Hand1**^+^**^/+^* hearts but fail to detect expression within *Hand1**^Δ^**^ST/**Δ**ST^* hearts (Figure 6C). Loss of the common exon in *Hand1**^Δ^**^ST/**Δ**ST^* hearts leads to loss of downstream exons as determined by qRT using primers against the last two exons of X4, X5, X6 (Figure 6B, P3, purple) indicate that loss of the common exon leads to loss of lncRNA transcript as detected by qPCR in *Hand1**^Δ^**^ST/**Δ**ST^* E10.5 hearts (Figure 6C).

## 4. Discussion

*Hand1* embryonic expression is regulated by a number of tissue-specific enhancers driving tissue-specific expression within post-migratory neural crest cells (NCC) within the sympathetic ganglia, the myocardium of the LV, and within the post-migratory NCC that populate the lower jaw and cardiac OFT [15,22,25]. *Hand1* expression is also observed within the ST of the developing embryo [13] and using a *Cre* recombinase *Hand1* knock-in allele [26] to delete *Hand2* reveals cardiac phenotypes of epicardial origin [13]. Transgenic reporter analysis identifies a conserved non-coding region that drives expression within the ST [15]. In this study, we delete this ST enhancer to determine its role in regulating *Hand1* expression within the ST and its role in PEO and epicardial formation.

Results show clearly that *Hand1**^Δ^**^ST/**Δ**ST^* mice are viable and without any discernable phenotypes. Moreover, *Hand1**^Δ^**^ST/LacZ^* compound heterozygotes are also viable and without any discernable phenotypes. Given that *Hand1* expression within the developing ST does not appear changed, we conclude that it is likely that a secondary ST enhancer, as yet unidentified, compensates in *Hand1**^Δ^**^ST/**Δ**ST^* mice. It is possible that the *Hand1* OFT/PA enhancer that lies just 3′ to the ST enhancer might also contribute to *Hand1* ST expression; however, previous transgenic reporter analysis does not support this possibility [15]. Interrogation of gene expression within *Hand1**^Δ^**^ST/**Δ**ST^* embryos for markers of the ST and PEO (*Tbx18*)*,* the epicardium (*Tcf21*), and established EPDCs (*Postn*) reveal no change in expression patterns supporting the lack of phenotype observed in these mice.

The ST and PEO, are incompletely marked by *Hand1* (Figure 3C) [13], suggesting that non-*Hand1* expressing cells might be sufficient to drive ST maturation during development. Additionally, non-PEO origin cells are known to contribute to the epicardium [27]. This heterogeneity of the PEO, and the non-PEO origin of the epicardium could explain a lack of phenotype in the ST enhancer knockout mice; however, given that *Hand1* expression patterns are not visibly changed, it is more likely that there is compensation for *Hand1* expression from a redundant enhancer.

One curious observation made in *Hand1**^Δ^**^ST/**Δ**ST^* embryos is that we observe a significant increase in *Hand1* cardiac expression. This could be the result of removing negative myocardial transcriptional inputs or the repositioning of *Hand1* OFT and LV enhancers within the open chromatin such that these enhancers become more efficient. The lack of phenotype in *Hand1**^Δ^**^ST/**Δ**ST^* embryos might also be due to the decrease in *Hand2* expression seen in *Hand1**^Δ^**^ST/**Δ**ST^* hearts (Figure 4M), suggesting a feedback regulatory mechanism between *Hand1* and *Hand2* expression levels *in vivo*. This compensatory mechanism might be heart-specific, as both *Hand1* and *Hand2* are significantly downregulated in the torsos within *Hand1**^Δ^**^ST/**Δ**ST^* embryos. What is clear is that the increase in *Hand1* cardiac transcripts does not result in observable phenotype, suggesting that there is broad toleration for different concentrations of *Hand1* transcript and/or that established mechanisms of post-translational regulation of HAND1 protein can mitigate deleterious gain-of-function phenotypes [28,29,30].

Finally, we show that a murine-specific predicted lncRNA family is actually expressed during embryonic development and a common lncRNA exon resides within the *Hand1* ST enhancer (Figure 6). The observed increase in *Hand1* expression within *Hand1**^Δ^**^ST/**Δ**ST^* hearts suggests that these lncRNAs might repress *Hand1*. Results show that deletion of the ST enhancer leads to a loss of expression of this novel lncRNA family; however, no phenotype is observed. Recent work from other groups has shown deletion of cardiac-specific lncRNAs is not required for embryo viability. Given many of these identified lncRNAs are not well conserved through evolution may reflect subtle roles in transcription, mRNA processing, or other molecular mechanisms that are species-specific [31].

Taken together, we conclude from this data that the *Hand1* ST enhancer is sufficient to drive ST expression during mouse embryogenesis [15] but is not necessary to maintain sufficient *Hand1* expression within the ST suggesting the existence of a redundant *Hand1* ST enhancer capable of driving normal epicardial development.
